# Nurse navigator. A concept analysis according to the Walker and Avant model

**DOI:** 10.17533/udea.iee.v44n1e06

**Published:** 2026-03-28

**Authors:** Maysa Mayran Chaves Moreira, Dayara Ainne de Sousa Araújo, Samara Dantas de Medeiros Diniz, Leilane Alice Moura da Silva, Maria Isabel da Conceição Dias Fernandes, Quenia Camille Soares Martins

**Affiliations:** 1 Nurse, M.Sc. in Health and Society. Email: maysa.mayran@gmail.com https://orcid.org/0000-0002-9576-9036 Universidade Federal do Rio Grande do Norte Health and Society Brazil maysa.mayran@gmail.com; 2 Nurse, Ph.D. student. Email: dayara-ainne@hotmail.com. Corresponding Author https://orcid.org/0000-0002-0593-2443 Universidade Federal do Rio Grande do Norte Brazil dayara-ainne@hotmail.com; 3 Nurse, M.Sc. student. Email: samaradantas1998@hotmail.com https://orcid.org/0000-0001-9418-0185 Universidade Federal do Rio Grande do Norte Brazil samaradantas1998@hotmail.com; 4 Nurse, B.Sc. Oncology Specialist. Email: leilanealice.ms05@gmail.com https://orcid.org/0000-0003-1930-0161 Universidade do Estado do Rio Grande do Norte Brazil leilanealice.ms05@gmail.com; 5 Nurse, Ph.D. Email: Isabel.dias@ufrn.br https://orcid.org/0000-0003-0569-5027 Universidade Federal do Rio Grande do Norte Brazil Isabel.dias@ufrn.br; 6 Nurse, Ph.D. Email: queniacamillesm@gmail.com https://orcid.org/0000-0002-4036-2423 Universidade Federal do Rio Grande do Norte Brazil queniacamillesm@gmail.com; 7 State University of Rio Grande do Norte, Mossoró, Rio Grande do Norte, Brazil Universidade Federal do Rio Grande do Norte State University of Rio Grande do Norte Mossoró Rio Grande do Norte Brazil; 8 Federal University of Rio Grande do Norte, Natal, Rio Grande do Norte, Brazil Universidade Federal do Rio Grande do Norte Federal University of Rio Grande do Norte Natal Rio Grande do Norte Brazil

**Keywords:** nurse-patient relationships, patient navigation, nursing care, continuity of patient care, health services., relaciones enfermero-paciente, navegación de pacientes, atención de enfermería, continuidad de la atención al paciente, servicios de salud., relações enfermeiro-paciente, navegação de pacientes, cuidados de enfermagem, continuidade da assistência ao paciente, serviços de saúde.

## Abstract

**Objective.:**

To analyze the concept of nurse navigator in light of the Walker and Avant model.

**Methods.:**

This is a concept analysis, based on the Walker and Avant model, operationalized through a scope review according to the JBI and PRISMA-ScR guidelines. The investigation follows other methodological stages for the construction and understanding of the “nurse navigator” concept.

**Results.:**

The attributes that prevailed were care management, health literacy, overcoming barriers, assertive communication, bonding, specialized knowledge, continued care, quality of care and patient integration into health systems. Regarding the most frequent antecedents, the following stand out: vulnerable populations, health disparities, communication gaps, delays and fragmentation of care, and low therapeutic adherence. The main consequences were the reduction of costs and mortality, reduction of hospitalizations and time spent in hospital, improvements in access to healthcare, adherence to treatment, quality of life and satisfaction with care.

**Conclusion.:**

The study defined the nurse navigator as the professional who manages care in a continuous way, capable of integrating it into the health system and strengthening the nurse-patient bond, reducing barriers and disparities, in addition to guaranteeing continuity and adherence to treatment.

## Resumen

**Objetivo.:**

Analizar el concepto de enfermero navegador a la luz del modelo de Walker y Avant.

**Métodos.:**

Se trata de un análisis de concepto, basado en el modelo de Walker y Avant, operacionalizado a través de una revisión de alcance según las directrices del JBI y PRISMA-ScR. La investigación siguió los ocho pasos metodológicos para la construcción y comprensión del concepto “enfermero navegador”.

**Resultados.:**

Los atributos que prevalecieron fueron la gestión del cuidado, la alfabetización en salud, la superación de barreras, la comunicación asertiva, el vínculo, el conocimiento especializado, la atención continua, la calidad de la atención y la integración del paciente a los sistemas de salud. En cuanto a los antecedentes más frecuentes, se destacaron: poblaciones vulnerables, disparidades en salud, fallas de comunicación, demoras y fragmentación de la atención, y baja adherencia terapéutica. Las principales consecuencias fueron la reducción de costos y mortalidad, reducción de hospitalizaciones y tiempo de hospitalización, mejoras en el acceso a la salud, adherencia al tratamiento, calidad de vida y satisfacción con la atención.

**Conclusión.:**

El estudio definió al enfermero navegador como el profesional que gestiona el cuidado de forma continua, capaz de integrarlo al sistema de salud y fortalecer el vínculo enfermero-paciente, reduciendo barreras y disparidades, además de garantizar la continuidad y la adhesión al tratamiento.

## Resumo

**Objetivo.:**

Analisar o conceito de enfermeiro navegador à luz do modelo de Walker e Avant.

**Métodos.:**

Trata-se de uma análise de conceito, baseada no modelo de Walker e Avant, operacionalizada mediante revisão de escopo conforme as diretrizes do JBI e PRISMA-ScR. A investigação seguiu as oito etapas metodológicas para à construção e compreensão do conceito “enfermeiro navegador”.

**Resultados.:**

Os atributos que prevaleceram foram a gestão do cuidado, letramento em saúde, superação de barreiras, comunicação assertiva, formação de vínculo, conhecimento especializado, cuidado contínuo, qualidade dos cuidados e integração do paciente nos sistemas de saúde. Quanto os antecedentes mais frequentes, destacaram-se: populações vulneráveis, disparidades em saúde, falhas na comunicação, atrasos e fragmentação do cuidado, e baixa adesão terapêutica. Os principais consequentes foram a redução de custos e da mortalidade, diminuição de internações e do tempo de hospitalização, melhorias no acesso à saúde, na adesão ao tratamento, na qualidade de vida e na satisfação do cuidado.

**Conclusão.:**

O estudo definiu o enfermeiro navegador como o profissional que gerencia o cuidado de forma contínua, capaz de integrá-los ao sistema de saúde e fortalecer o vínculo enfermeiro-paciente, reduzindo barreiras e disparidades, além de garantir a continuidade e adesão do tratamento.

## Introduction

Noncommunicable chronic diseases (NCDs) are the leading causes of illness and mortality globally, generating several negative impacts on quality of life, such as disabilities, increased morbidity, higher costs for health systems, and direct harm to the socioeconomic development of countries.^1^^,^^2^ Furthermore, the growth in the incidence of these diseases is associated with changes in habits and lifestyles, population aging, as well as socioeconomic disparities and inequalities in access to health services.^3^ As a consequence, health indicators are worse in countries and populations with social vulnerability, as are the social determinants and risk factors of chronic diseases in individuals with low education and income. Given this, many health professionals are still not prepared to meet the particular needs of this user profile.^4^^,^^5^ In this context, in order to expedite the diagnosis and continuity of treatment of chronic diseases, the American physician Harold Freeman, in 1990, developed the idea of ​​patient navigation (PN). This process facilitates patients' access to healthcare systems and services, aiming to overcome the socioeconomic, cultural, financial, bureaucratic, and psychological barriers to treatment.^6^

Thus, in collaboration with the American Cancer Society (ACS), the first navigation program, called the Patient Navigator Program, was developed at Harlem Hospital in New York. The program was designed to identify the difficulties faced by patients in accessing cancer treatment, from screening to palliative care.^7^ From this perspective, the patient navigator guides and assists individuals diagnosed with or suspected of having a chronic disease - or patients at risk of negative clinical outcomes - in “navigating” the healthcare system. In this context, a navigation program represents the integration between the care process for chronic patients and the different levels of healthcare, through the navigator. This increases the likelihood that patients will adhere to the recommended treatment, while also reducing clinical barriers and health disparities.^8^^,^^9^ Currently, PN programs are continuously evolving and are aimed at patients with various types of chronic diseases, especially oncological conditions, in both primary care services and high-complexity settings. Thus, they are widely adopted in the United States, seeking to identify and overcome barriers throughout the therapeutic journey, as well as contribute to reducing delays in accessing healthcare services. Furthermore, evidence indicates that their implementation improves access to treatment and supports continuity of the therapeutic process.^10^^,^^11^

Given its numerous benefits, the navigation practice has expanded to other care settings and is carried out by different professionals and lay individuals; however, studies show that the nurse navigator (NN) plays a central role in providing individualized care to patients and families, promoting access to healthcare services and ensuring quality of care at all stages of the care process.^7^^,^^12^^,^^13^ The term Nurse Navigator refers to professionals with clinical experience and specialized knowledge, who guide his/her care based on a variety of social, economic, and cultural aspects. In this way, nurses in this form of care guide families, caregivers, and patients throughout the treatment, offering information, support, comprehensive care, and empowerment for shared decision-making with the multidisciplinary team. ^14^^,^^15^

In Brazil, few institutions have implemented this program, and its roles, importance, and specificities are still not well defined.^16^ Therefore, the concept of the Nurse Navigator must be understood in order to contribute to the growth of this professional category, expand opportunities within healthcare services, and strengthen holistic and patient-centered care. Thus, a gap was identified in the literature regarding the concept of the nurse navigator, making it necessary to understand its definition as well as the elements that compose it. This would allow for the standardization of the care model so that it can be operationalized to ensure safe, high quality, and targeted care. It is noteworthy that the concept promotes a phenomenon that, once defined, contributes to the development of evidence-based practice.^16^ Therefore, this study aims to analyze the concept of the nurse navigator in light of the Walker and Avant model.

## Methods

### Study design

This is a concept analysis based on the Walker and Avant model (2019), operationalized through a scope review, carried out between September 2024 and May 2025.^17^^,^^18^ Concept analysis aims to standardize the description of a phenomenon and allow for effective communication, thus reducing vague terminology, making it more operational in theory and practice. In this study, the Walker and Avant model was used. To this end, the investigation was structured in eight stages, namely: I) selecting the concept; II) outlining the goals and objectives of the analysis; III) determining the possibilities of using the concept; IV) determining the attributes; V) proposing/using a model case; VI) constructing additional case(s); VII) detecting antecedents and consequences; and VIII) defining the empirical references.^17^ In this way, the concept of "nurse navigator" was established, with the aim of analyzing the nurse navigator's intervention in the lines of care in health care. Furthermore, to support the conceptual analysis, a scoping review was used according to the recommendations of the Joanna Briggs Institute (JBI), based on the Preferred Reporting Items for Systematic Reviews and Meta-Analyses Extension for Scoping Reviews (PRISMA-ScR). The study protocol was registered in the Open Science Framework (DOI: 10.17605/OSF.IO/6AB7K).^18^^-^^20^

### Protocol of study

The scoping review was chosen to map studies of diverse natures and incorporate the practical applicability of results found in the literature, to provide a holistic understanding of a phenomenon. Therefore, a research protocol was used, composed of the following stages: theme, objectives, research question, identification of relevant studies through literature search, selection of studies with establishment of eligibility criteria; mapping, data extraction and presentation of results.^18^ The guiding question was based on the PCC strategy - P (population), C (concept) and C (context). Thus, the population consisted of nurses, the concept of patient navigation, and the context corresponded to the care pathways in health care. Therefore, the main question was: “What is the definition of the nurse navigator concept?” The subsequent questions of the study were: “What are the attributes of the nurse navigator concept in the care pathways in health care? What are the antecedents and consequences of the nurse navigator concept?”

Data collection was carried out through electronic searches in the following databases: SCOPUS, Web of Science, Virtual Health Library (VHL), Cumulative Index to Nursing and Allied Health Literature (CINAHL), Cochrane Library, and MEDLINE/PubMed. Grey literature searches were conducted using Google Scholar®. The content was accessed via the Federated Academic Community (CAFe), through the portal of the Coordination for the Improvement of Higher Education Personnel (CAPES). For the databases, an advanced search was performed using the Health Sciences Descriptors (DeCS) and their respective Medical Subject Headings (MeSH): “Relações Enfermeiro-Paciente”; “Nurse-Patient Relations”; “Navegação de Pacientes”; “Patient Navigation”; “Continuidade da Assistência ao Paciente”; “Continuity of Patient Care”. The Boolean operators “AND” and “OR” were used for the following intersection: “Relações Enfermeiro-Paciente” OR “Nurse-Patient Relations” AND “Navegação de Pacientes” OR “Patient Navigation” AND “Continuidade da Assistência ao Paciente” OR “Continuity of Patient Care”.

For the selection of studies, the following inclusion criteria were used: full-text publications available in the databases that addressed patient navigation carried out by nurses. The exclusion criteria were: editorials, letters to the editor, abstracts, duplicate works, expert opinions, protocols, correspondences, reviews, and book chapters. To ensure the comprehensiveness and diversity of the documentary information, no eligibility criteria were established regarding the year of publication or the language of the studies analyzed.

### Data organization and analysis

Two reviewers conducted the selection of studies; the initial screening was performed through the reading of titles and abstracts, followed by a full reading of the selected studies. Duplicate studies were counted only once, and those that did not meet the eligibility criteria were excluded. For the evaluation of the studies, the following indicators were used: title; country; year of publication; language; attributes of the concept(s) presented on the topic; characteristics/particularities regarding the Nurse Navigator; aspects that contributed to the proximity and emergence of the term Nurse Navigator; and consequences resulting from the application of the Nurse Navigator concept. The data were organized and tabulated in spreadsheets in Microsoft Excel 2016 and presented in tables and figures to facilitate the interpretation and understanding of the concept investigated, according to the Walker and Avant framework.^17^

Regarding the level of evidence, the classification of the Joanna Briggs Institute (JBI) Collaborating Centre was adopted. The studies were assessed as follows: Level 1 - Evidence obtained from a systematic review of randomized controlled clinical trials; Level 2 - Evidence obtained from a randomized controlled clinical trial; Level 3.1 - Evidence obtained from well-designed controlled clinical trials without randomization; Level 3.2 - Evidence obtained from well-designed cohort or case-control studies; Level 3.3 - Evidence obtained from multiple time series, with or without intervention, and dramatic results from uncontrolled experiments; and Level 4 - Opinions of respected authorities based on clinical criteria and experience, descriptive studies, or reports from expert committees.^21^

## Results

**Figure 1:**
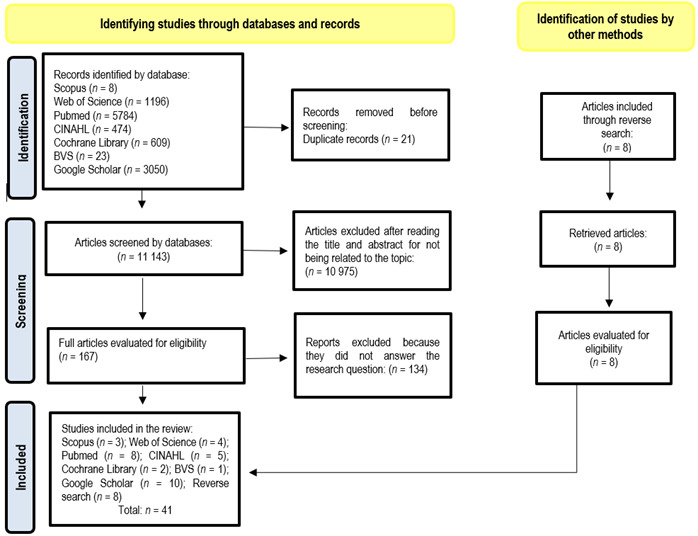
PRISMA 2020 Flowchart, adapted for Scope Review.

The initial search identified 11,144 studies. After reading the titles and abstracts, performing a full analysis, and rigorously observing the inclusion and exclusion criteria, a final sample of 41 articles was obtained, as described in Figure 1 regarding the selection process.

From the 41 studies that comprised the final sample, it was evident that the highest number of publications occurred in the last five years (51.2%), with North America standing out as the continent with the highest number of studies on the topic (48.7%), followed by South America with 26.1% of publications. Regarding language, 82.9% were published in English. 

### Identification of the uses of the concept

For this analysis, the concept of "nurse navigator" was selected, allowing for the identification of essential aspects for understanding the term. The studies are characterized in Table 1.

**Table 1:** Characterization of the studies according to authors, year of publication, type of study, identification of the use of the "Nurse Navigator" concept, and level of evidence

Author	Year of publication	Type of study	Identification of the uses of the concept “Nurse Navigator”	Level of evidence
Sullivan KA *et al*.^22^	2015	Cohort	Coordination of patient-centered care assists patients in accessing resources, including medical care and community services, and provides enhanced support commensurate with a higher level of education and greater familiarity with conditions and treatments. Performed by advanced practice nurses.	3.2
Thygesen MK *et al*.^23^	2012	Phenomenology	It provides personalized information about the disease. It manages cases, thus serving as an open line of communication with patients and continuous contact with the health service.	*
Horner K *et al*.^24^	2013	Clinical Trial	It provides targeted care management with proactive care. Monitors and manages symptoms and provides psychosocial support.	2
Newton JC *et al*.^25^	2022	Phenomenology	This study did not present the concept.	*
Soin A *et al*.^26^	2022	Cohort	They are trained professionals who can help patients identify their health goals and overcome barriers to achieving them. They ensure consistency within the team and maximize patient engagement to provide continuous care.	3.2
Manderson B *et al*.^27^	2011	Systematic review	It contributes to improving access to and coordination of patient care within the healthcare system.	1
McBrien KA *et al*.^28^	2018	Systematic review	To help people with chronic illnesses navigate and access healthcare services. Patient navigators most often provide education about tests, treatments, and psychosocial support.	1
Chan RJ *et al*.^29^	2023	Systematic review	The nurse navigators facilitate continuity of care, teach and guide, develop supportive and therapeutic relationships. They provide access to resources and services and empower patients to make informed decisions. This care coordinator role requires skills in administrative leadership, collaboration, and support from all stakeholders.	1
Katerenchuk J; Santos AS^30^	2021	Integrative review	Nurse navigators are involved in coordinating care. They are proficient professionals in this role and possess the training and experience to use critical thinking and decision-making skills relevant to the evolution of care processes.	4
Baileys K *et al*.^31^	2018	Integrative review	The nurse navigator coordinates the overall care of patients throughout distinct phases of treatment, removes barriers to care, and provides timely access.	4
Gordils-Perez J *et al*.^32^	2017	Cohort	The nurse navigator coordinates care, communication, education, and professional roles. In addition, he/she provides specialized care.	3.2
Lubejko BG *et al*.^33^	2019	Systematic review	This study did not present the concept.	1
Lim H *et al*.^34^	2021	Quasi Experimental study	Nurse navigators are responsible for moving patients along the continuum of care to promote patient integration into the healthcare system.	3.1
Rodrigues RL *et al*.^6^	2021	Integrative review	These oncology professionals use their specialized knowledge, clinical experience, and skills to provide patients with care focused on physical, social, and emotional aspects. They guide patients, families, and caregivers in making joint decisions with the multidisciplinary team responsible for treatment. In addition to managing treatment, nurse navigators also provide information related to it.	4
Pautasso FF *et al*.^35^	2018	Integrative review	The domain or categories of tasks derived from the needs analysis included the coordination of services and the identification of support networks.	4
Guha C *et al*.^36^	2022	Clinical Trial	The nurse navigator contributes to the quality of care provided and to the overall well-being of the patient.	2
Duzova US; Can G^37^	2021	Clinical Trial	Providing care through the integration of health systems creates a continuous flow of care, eliminates barriers that impede access to health services, and provides connectivity between health systems.	2
Pautasso FF *et al*.^15^	2020	Convergent Healthcare Research	The nurse navigator provides effective support to patients, delivers information, and manages the complexity of diagnosis and treatment in conjunction with all members of the multidisciplinary team.	3.3
Roque AC *et al*.^10^	2023	Descriptive	These professionals utilize their specialized knowledge, clinical experience, and skills to provide patients with care focused on physical, social, and emotional barriers. They guide patients, families, and caregivers in making joint decisions with the multidisciplinary team responsible for treatment. They oversee the entire treatment process, providing information and support.	4
Lima MERF *et al*.^38^	2021	Integrative review	The nurse navigator assists in understanding the diagnosis, treatment, guidance on home care, consultations, and examinations. Furthermore, he/she can facilitates screening, diagnosis, treatment, and support throughout the continuous care process.	4
Anjos TR^39^	2023	Scope review	This study did not present the concept.	4
Roque AC *et al*.^16^	2022	Integrative review	The nurse's role focuses on coordinating the continuity of care and monitoring the patient's self-care.	4
Lima FC *et al*.^40^	2025	Integrative review	These professionals provide patient-centered care with timely access. Nurse navigators play a critical role in cancer screening and service coordination. They are often the primary point of contact for patients accessing the healthcare system.	4
Doerfler-Evans RE^41^	2016	Narrative Review	The nurse navigator provides detailed education. He acts as a communication bridge between patients and the provider.	4
Shockney LD *et al*.^42^	2021	Scope review	The nurse navigator coordinates and monitors the organization of the patient's journey, in addition to facilitating the progress of treatment and promoting integration among the various professionals involved in the treatment.	4
Pinheiro DO *et al*.^43^	2023	Integrative review	The nurse navigator guides the care process in a concise and cohesive manner, helping the individual with cancer through the entire journey of diagnosis and treatment. This includes assisting with acceptance, treatment, procedures, adverse reactions, home care, consultations and examinations, support for family members, among other responsibilities of this professional.	4
Borchartt DB *et al*.^44^	2022	Integrative review	The nurse navigator acts as a coordinator within the healthcare system, supporting and monitoring the patient throughout the continuity of care during their illness.	4
Adler G *et al*.^45^	2019	Cohort	This professional facilitates timely and quality patient care by resolving abnormal screening studies.	3.2
De La Cruz I *et al*.^46^	2014	Clinical Trial	This study did not present the concept.	2
Slind LM *et al*.^47^	2016	Cross sectional study	The Nurse Navigator provides personalized assistance, support, and resources.	3.3
Collier S *et al*.^48^	2022	Cohort	This professional addresses barriers to care, educates people about their health condition, and regularly contacts people about their treatment status.	3.2
Bush ML *et al*.^49^	2018	Systematic review	The nurse navigator assists patients in assessing and mitigating personal and environmental factors to promote healthy behaviors.	1
Yatim F *et al*.^50^	2017	Mixed methods	The nurse navigator reduces barriers to patient access to care and improves coordination of the clinical pathway.	*
Gervès-Pinquié C *et al*.^51^	2017	Clinical Trial	Nurse navigators are responsible for improving care transitions, providing information from prevention to treatment, assisting patients with medical paperwork, facilitating patient-professional communication, scheduling appointments, addressing transportation issues, and mobilizing the patient's financial resources.	2
Budde H *et al*.^52^	2021	Systematic review	The nurse navigator improves access to treatment services for minority groups. Patient navigation can encompass various tasks throughout the continuum of care, including education, outreach, facilitating communication, and end-of-life care.	1
Heritage B *et al.*^53^	2020	Cross sectional study	The Nurse Navigator coordinates patients throughout a care journey, works with a multidisciplinary team, involves all stakeholders (community and acute care), improves patient outcomes through evidence-based practice, and facilitates systems improvement as a clinical leader and change agent.	3.3
Wong CL *et al*.^54^	2024	Scope review	They are responsible for coordination; they play an essential role in the transition of pediatric patients, and help reduce the gap between child and adult care services.	4
Howitt L *et al*.(^55^)	2024	Systematic review	The responsible nurse assesses symptoms, provides spiritual and social support, discusses treatment goals and preferences, and makes referrals to other providers and services when necessary.	1
Jesus TS *et al*.^56^	2024	Systematic review	This study did not present the concept.	1
Pratt-Chapman M *et al*.^57^	2011	Integrative review	This study did not present the concept.	4
Lopez D *et al*.^58^	2019	Narrative Review	This study did not present the concept.	4

* Level of evidence that does not classify in qualitative studies

Regarding the attributes that deal with the terms that assist in the characterization and construction of the definition of the concept under study. Furthermore, regarding the antecedents, terms or phrases responsible for contributing to the emergence of the concept, and the consequents, which address the results arising from the concept, these are presented in Table 2.

**Table 2:** Presentation of the Attributes, Antecedents, Consequents, and Definition of the "Navigator Nurse" concept


Attributes	Antecedents	Consequents
Care management He promotes health literacy He overcomes care barriers Assertive communication He integrates the health system Bond/relationship building Continuous care Specialized knowledge Quality of care	Vulnerable populations; Patient passivity; Ethnic disparity; Socioeconomic disparity; Cultural disparity; Geographic disparity; Communication failure; Delay in care; Delay in diagnosis; Delay in treatment; Fragmented care; Difficulty accessing healthcare services; Treatment barriers; Low adherence; Multiple comorbidities;	Reduction of healthcare costs Reduction of mortality It reduces barriers to care It reduces hospital admission It reduces length of hospital stay It improves access to healthcare services It improves quality of life It improves satisfaction with care It improves treatment adherence It contributes to self-management Effective communication Multiprofessional coordination Promotes health literacy Establishment of a nurse-patient bond

Definition	The Nurse Navigator is defined as a professional with specialized knowledge who manages care continuously, capable of integrating the user's needs into the health network. In this way, they strengthen the nurse-patient bond, overcome treatment barriers, and promote health literacy.	

### Identifying a Model Case and a Contrary Case

Regarding the model case and the contrary case, fictitious and illustrative cases were constructed for a better understanding of the concept under analysis.

Model Case for “Nurse Navigator. In the model case, we have MMC, 56 years old, married, mother of three, a homemaker, and a native of Natal. She reports systemic arterial hypertension, with irregular use of medications and a diet high in salt, lipids, and carbohydrates. She denies smoking and alcohol use. She comes to the outpatient clinic of the hospital unit for an appointment, accompanied by her daughter, to receive the biopsy result after undergoing a screening mammogram, which showed a BIRADS 4 result in the right breast. The histopathological diagnosis revealed ductal carcinoma in situ (DCIS) of the breast. The medical team informs her of the diagnosis of breast cancer with conservative treatment, involving a sector resection with margin enlargement and sentinel lymph node management, followed by adjuvant radiotherapy. Immediately afterward, the patient is referred for a consultation with the Nurse Navigator, a specialist professional working within the oncology network. During this consultation, the Nurse Navigator identified some vulnerabilities and difficulties faced by the patient and her companion, such as transportation challenges for treatment. As a result, the nurse navigator contacts the social services department to resolve this issue. Throughout the entire treatment process, this professional was present-either in person or by phone-allowing for the development of bond, improved multiprofessional coordination, and reduced healthcare costs. Furthermore, the Nurse Navigator promoted health literacy and self-care for MMC, contributing to the continuity and quality of the care provided.

Contrary Case for “Nurse Navigator.” MMC, 56 years old, married, mother of three, homemaker, born in Natal. She reports systemic arterial hypertension, with irregular use of medications and a diet high in salt, lipids, and carbohydrates. She denies smoking and alcohol use. She comes to the outpatient clinic of the hospital unit for a consultation, accompanied by her daughter, to receive the biopsy result after undergoing a screening mammogram that showed a BI-RADS 4 result in the right breast. The histopathological diagnosis was ductal carcinoma in situ (DCIS) of the breast. The medical team informs her of the breast cancer diagnosis with a conservative treatment plan consisting of a sector resection with widened surgical margins and sentinel lymph node management, followed by complementary adjuvant radiotherapy. Immediately afterward, the patient is referred for a nursing consultation to receive further guidance regarding the beginning of treatment, which she is unable to fully comprehend due to the large amount of information. Even so, she begins treatment feeling very worried and distressed, without assurance of treatment continuity because of social and economic issues. Therefore, based on the possible uses of the concept, its critical attributes, and the development of the cases, a definition was constructed to be applied to the concept of “Nurse Navigator,” namely: a professional with specialized knowledge who manages care continuously and is capable of integrating the user’s needs within the healthcare network. In doing so, they strengthen the nurse-patient bond, overcome treatment barriers, and promote health literacy.

### Identification of Empirical Referents

Furthermore, to support the concept analysis-given that it is an emerging topic in the field of nursing-the literature included a study that addressed the measurement of the need for patient navigation by nurses, entitled *“Nurse Navigator: development of a program for Brazil”*, which developed a Navigation Needs Assessment Scale (NNAS*) with a minimum score of six points (the patient has no need for navigation) and a maximum score of 17 points (there is a need for navigation performed by the nurse).^15^

## Discussion

Although navigation programs already exist in some countries, this practice is still relatively recent. Even with its more consolidated presence in North American countries, significant challenges persist regarding the definition of processes and functions, as well as the training and qualification of navigators. However, these programs share the same main objective.^24^^,^^35^ Nurse navigators are trained to understand the impact of a diagnosis on patients and their families. Due to their training, they possess the necessary skills to act directly on treatment barriers, as they accompany patients throughout their journey, and have a greater chance of forming a bond. Therefore, these professionals have maintained the position of navigators, especially in oncology, who are part of a multidisciplinary team and focus their actions on achieving the desired results.^15^^,^^35^^,^^43^

Regarding the main attributes identified in the study - words or expressions frequently used in the literature to characterize the concept - it is observed that these can be adapted according to the concept presented. This identified aspects that contribute to understanding the role of the Nurse Navigator, such as: overcoming barriers to treatment, managing care, building relationships, assertive communication, continuous care, specialized knowledge, promoting health literacy, and quality of care. Through the evaluation of the social determinants of health of a population, such as knowledge of social, political, economic, and cultural aspects, the Nurse Navigator becomes more efficient and targeted. In this way, in addition to contributing to overcoming barriers to care, he/she identifies the patients who most need this type of monitoring and ensures continuity of care for these patients. Furthermore, he/she improves access to healthcare services for all groups, including minority groups.^48^^,^^52^

It became evident that the nurse navigator fulfills multiple functions, but one of the main ones is serving as the patient’s primary point of contact with health services and eliminating socioeconomic, cultural, racial, and ethnic barriers that are often present in healthcare systems and hinder access, continuity, and adherence to recommended treatment. Thus, the relationship between the navigator and the patient is fundamental. Therefore, it is important for the nurse navigator to develop a horizontal and trusting relationship with the patient, helping to shape transformative agents within his care context.^10^^,^^26^^,^^31^^,^^40^ From this perspective, studies highlight that the nurse navigator may encompass multitasking throughout the continuum of care, including outreach, facilitation of communication, and education.^52^ Likewise, this professional has the profile of a natural educator, being responsible for improving care transitions, providing personalized and preventive information about the patient’s health condition, and regularly contacting individuals regarding their treatment status. Nurse navigators are also able to facilitate screening, diagnosis, treatment, and support throughout the continuum of care, in coordination with the multidisciplinary team.(^23,40,48,51)^

The nurse navigator’s role provides access to information that may influence future decisions and contribute to guiding patient care. One study showed that the work of these professionals ensured significant improvement, enabling patients diagnosed with neoplasms to begin their systemic treatment earlier.^6^^,^^38^ The navigator assists patients in coordinating all stages of care, such as support with medical documentation, scheduling appointments, mobilizing financial resources and transportation matters, as well as facilitating patient-professional communication.^44^^,^^51^

Care management is provided through proactive service. In addition, clinically, the NN will be able to monitor and manage patient symptoms, assess and assist psychosocial, physical, environmental, and spiritual factors, in order to promote healthy behaviors.^24^^,^^49^^,^^55^ Patient-centered care management performed by advanced practice nurses facilitates access to health resources and community support, providing appropriate assistance aligned with patients’ needs and level of understanding. By working with multidisciplinary teams and following the patient journey, the nurse navigator contributes to better clinical outcomes through evidence-based practice, thus promoting changes in health services and assuming the role of clinical leader and transformation agent.^22^^,^^53^ To that end, a barrier identified for nurse navigators is the lack of knowledge regarding their role, manifesting as limited experience with patient navigation and the absence of a defined standard for their responsibilities. Nonetheless, the professional contributes to effective communication and accompanies the adaptation process; therefore, they can encourage self-care and ensure access to all available resources, whether for treatment or for social needs that reduce health disparities.^32^

An analysis of the antecedents and consequences of the nurse-navigator concept identifies his relevance in overcoming various challenges present in healthcare, especially in vulnerable populations. Among the main antecedents identified are: ethnic, socioeconomic, cultural, and geographic disparities, barriers to treatment access, fragmentation of care, communication failures, and delays in care, diagnosis, and treatment. In these situations, the nurse-navigator acts as a fundamental agent for care coordination, thus promoting effective communication, multidisciplinary collaboration, and the formation of a bond with the patient. As a consequence of this process, significant improvements are observed, such as a reduction in hospitalizations and length of stay, decreased healthcare costs, increased adherence to treatment and satisfaction with care, as well as the promotion of health literacy, self-management, and consequently, improved quality of life and reduced mortality. Therefore, the findings reinforce the strategic role of the nurse navigator in improving the quality of care and addressing health inequities.

Identifying empirical benchmarks is also crucial for consolidating the concept of patient navigation in nursing, especially since it is an emerging topic. Several studies contribute significantly by developing the Navigation Needs Assessment Scale (NNAS), which allows for the objective identification of when intervention by the nurse navigator is necessary. This tool supports clinical decision-making and reinforces the importance of validated instruments in clinical practice.^15^

In short, the presence of the navigation process carried out by the nurse professional increases patient satisfaction and overall quality of life by supporting decision-making, offering emotional support, reducing distress, and helping to resolve issues arising from the treatment of chronic diseases. As a result, users, caregivers, and family members achieve better outcomes in coordination, cost-effectiveness, and adherence to care.^32^ The present study showed limitations related to restricted access to some studies, such as only having access to abstracts, which could have enriched the data and strengthened the information derived from the final sample of this research. Furthermore, it was observed that there is a limited number of articles addressing a standardized concept of the nurse navigator, highlighting the importance of original studies on the topic. This finding not only emerged during the development of the research but is also supported by the literature, which demonstrates the need for scientific works that define more consistent conceptual standards and guidelines concerning the role and responsibilities of the nurse navigator at a global level.^59^^,^^60^^,^^61^

Conclusion:

The final sample enabled the analysis and identification of the concept of the “Nurse Navigator” through its attributes, cases, antecedents, and consequences, making it possible to outline the aspects necessary for the operationalization of the concept and to achieve the proposed objective. Thus, the “Nurse Navigator” is defined as a professional with specialized knowledge who manages care continuously and is capable of integrating users’ needs within the healthcare network. In this way, the role strengthens the nurse-patient bond, overcomes treatment barriers, and promotes health literacy. Therefore, this study contributed to broadening and enhancing the understanding of the nurse navigator concept, so it can be better applied in the systematization of nursing care and in the participation of the multidisciplinary team.
